# Applications of Photopic Negative Response: A Narrative Review

**DOI:** 10.3390/jcm15093527

**Published:** 2026-05-05

**Authors:** Minzhong Yu, Nara Shakaki, Anas Bakdalieh

**Affiliations:** 1 Department of Ophthalmology and Visual Sciences, University Hospitals Eye Institute, Case Western Reserve University, Cleveland, OH 44106, USA; 2 Cole Eye Institute, Cleveland Clinic Foundation, Cleveland, OH 44106, USA; 3 Department of Ophthalmology, Cleveland Clinic Lerner College of Medicine of Case Western Reserve University, Cleveland, OH 44195, USA; 4 College of Medicine, Northeast Ohio Medical University, Rootstown, OH 44272, USA; nshakaki@neomed.edu (N.S.); abakdalieh@neomed.edu (A.B.)

**Keywords:** photopic negative response, electroretinography, glaucoma, optic neuropathy, intracranial hypertension, retinal ischemia

## Abstract

**Background**: The photopic negative response (PhNR) of the full-field electroretinogram is a retinal ganglion cell-weighted functional signal increasingly proposed as a clinical biomarker. Despite extensive study across ocular and systemic diseases, its precise clinical role and incremental value remain incompletely established. **Methods**: This narrative review synthesizes key human studies of the photopic negative response, with emphasis on physiological basis, recording methodology, and clinical contexts in which PhNR may provide added functional insight. **Results**: In glaucoma, PhNR provides an objective measure of retinal ganglion cell dysfunction that correlates moderately with optical coherence tomography (OCT)-derived structural loss and visual field indices, but with substantial inter-individual variability. Its greatest clinical utility lies in early disease detection, cross-sectional functional assessment, and documenting short-term functional changes following intraocular pressure reduction, rather than longitudinal progression monitoring. Beyond glaucoma, PhNR reveals inner retinal dysfunction in systemic and genetic conditions, particularly idiopathic intracranial hypertension and diabetes, where retinal ganglion cells may reflect broader neurological or metabolic stress. **Conclusions**: PhNR is best viewed not as a standalone diagnostic or progression tool, but as a complementary functional biomarker that adds objective insight when structural imaging or psychophysical testing is limited or discordant. Its role aligns closely with the retina’s emerging function as a mirror of systemic and genetic disease, provided recordings are standardized and results interpreted cautiously.

## 1. Introduction

**Figure 1:**
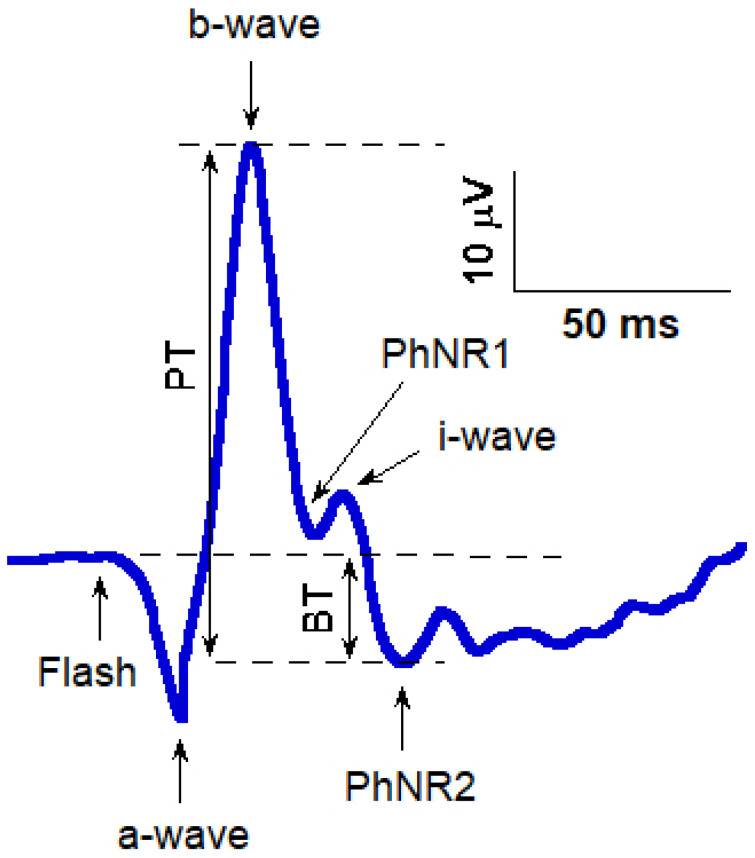
Schematic illustration of a photopic negative response (PhNR) waveform. Following the flash stimulus, the initial negative deflection represents the a-wave, primarily reflecting photoreceptor activity, followed by the positive b-wave, generated largely by bipolar and Müller cell activity. An i-wave is visible on the descending limb of the b-wave. The PhNR is shown with two commonly used troughs: PhNR1, occurring shortly after the i-wave, and PhNR2, the later and typically deeper negative trough; both are associated with retinal ganglion cell function. Amplitudes are indicated as PT (peak-to-trough, from b-wave peak to PhNR trough) and BT (baseline-to-trough, from pre-stimulus baseline to the PhNR trough). Calibration bars represent 10 μV vertically and 50 ms horizontally.

The clinical electroretinogram (ERG) has long served as an objective measure of overall retinal physiology. Under light-adapted conditions, the photopic negative response (PhNR) emerges after the cone b-wave as a slow negative trough that is typically measured either from the pre-flash baseline to trough (BT) or from the b-wave peak to trough (PT) (Figure 1). Its generators are closely linked to retinal ganglion cell (RGC) activity, as evidenced by pharmacology, primate models, and human disease cohorts. Foundational work demonstrated that experimental glaucoma in macaques selectively attenuates the PhNR, a finding quickly echoed in primary open-angle glaucoma (POAG) patients [1]. These observations positioned PhNR as a functional complement to structure-based diagnostics (OCT) and psychophysics (standard automated perimetry, SAP), especially for early disease and treatment response [2].

Recognizing its translational potential, the International Society for Clinical Electrophysiology of Vision (ISCEV) published an extended protocol to harmonize PhNR recordings, detailing stimulus conditions, electrode considerations, and reporting conventions. This standardization catalyzed a surge in clinical applications and comparative studies across diseases affecting the inner retina [3].

In most clinical and research protocols, the PhNR is quantified using the second negative trough after the b-wave, which corresponds to the prominent trough occurring after the small positive i-wave. This post-i-wave trough (termed PhNR2) is generally more easily distinguished, because it is separated from the rising phase of the b-wave and is less susceptible to overlap with earlier components. However, an earlier negative deflection, PhNR1, between the b-wave and the i-wave can also be measured and is often more consistently detectable when the post-i-wave segment is noisy or when the i-wave itself is reduced in certain disease states. Although PhNR2 has traditionally been regarded as the canonical PhNR metric, PhNR1 and PhNR2 amplitudes show a strong linear relationship and are believed to reflect similar RGC-mediated activity. Therefore, PhNR1 may serve as a reliable alternative when PhNR2 is ambiguous, but standardized criteria for identifying each trough are essential to enable valid comparisons across studies and accurate assessment of RGC function [4]. Practical criteria for their selection are described in the Methods. A summary of key PhNR characteristics, measurement considerations, and clinical applications is provided in Table 1.

This review does not aim to provide a formal diagnostic accuracy evaluation or biomarker validation study. Rather, it provides a clinically oriented narrative synthesis focusing on the PhNR as a complementary RGC-weighted functional biomarker. Specifically, we examine its roles in cross-sectional functional assessment, early detection of RGC dysfunction, and short-term responsiveness to therapeutic interventions across ocular and systemic disease contexts.

## 2. Methods

### 2.1. Search Strategy

This article is a narrative review based on the authors’ assessment of the clinical and experimental literature on PhNR. The literature was surveyed primarily via PubMed through March 2026 using the term “photopic negative response”. Only English-language publications were included. Rather than employing formal quality scoring or meta-analysis, emphasis was placed on studies with clear clinical relevance, representative sample sizes, and methodological rigor, based on study design, sample size, and reproducibility. The goal was to provide interpretive synthesis rather than exhaustive enumeration of all available reports.

### 2.2. Study Prioritization

Given the heterogeneity of designs, a narrative synthesis approach was adopted. Studies were prioritized using the following hierarchy: (1) Investigated the physiological basis of PhNR or its recording methodology. (2) Reported clinical applications of PhNR in human subjects, including glaucoma, optic neuropathies, retinal ischemic disorders, or systemic diseases affecting the retina. (3) Described technical protocols or guidelines relevant to PhNR (e.g., ISCEV standards). (4) Provided comparative data between PhNR and other diagnostic modalities (e.g., OCT, PERG, visual fields).

### 2.3. Exclusion Criteria

Studies were excluded if they:

(1) Were abstracts without peer-reviewed full text.
(2) Focused solely on outer retinal physiology without analysis of PhNR.
(3) Were isolated case reports with <3 patients unless they provided unique mechanistic or methodological insights.

### 2.4. Data Extraction and Synthesis

Two independent reviewers (M.Y. and A.B.) screened titles and abstracts to identify potentially relevant studies. Disagreements between reviewers were resolved through discussion and consensus. Full texts were then evaluated for inclusion. Extracted information included study design, patient population, recording methodology, PhNR measurement parameters, and key outcomes.

Given the heterogeneity of study designs and outcome measures, no quantitative meta-analysis was performed. Instead, a qualitative synthesis was conducted. Studies were organized into thematic domains:

(1) Physiological basis of PhNR
(2) Technical considerations and standardization
(3) Clinical applications in glaucoma and optic neuropathies
(4) Applications in vascular and systemic disease
(5) Emerging directions, including mfPhNR, handheld devices, and machine learning

Emphasis was placed on studies with robust methodology, larger sample sizes, and clear clinical relevance. As a narrative review, this synthesis may be subject to selection bias, and no formal risk-of-bias assessment or quantitative synthesis was performed.

## 3. Physiological Basis: What the PhNR Measures

PhNR primarily reflects the net activity of RGCs and their postsynaptic circuits. In vivo and experimental models support this attribution: RGC injury or blockade reduces PhNR with relative preservation of earlier outer-retinal components; conversely, manipulations sparing RGCs affect the PhNR less. In POAG and experimental glaucoma, selective PhNR loss occurs alongside RNFL thinning, reinforcing a structure-function link [2].

While the peak time of the PhNR is less commonly emphasized than amplitude (given its shallow, prolonged trough), the ISCEV protocol highlights that amplitude criteria and stimulus design (e.g., narrowband red flashes on blue backgrounds) critically shape the recorded waveform. The PhNR is modestly age-dependent, reinforcing the need for age-matched norms and device-specific reference ranges [3].

## 4. Recording and Analysis: Practical Considerations

PhNR amplitude and implicit time are influenced by stimulus configuration, background adaptation, and electrode type, underscoring the importance of protocol consistency across laboratories [5].

### 4.1. Stimulus and Adaptation

ISCEV’s extended protocol recommends photopic adaptation, narrowband red flashes on a blue background (RB) or, in some workflows, white-on-white (WW) flashes, with careful specification of luminance/energy and inter-flash timing. RB often yields robust PhNR signals with good separation from the i-wave; WW can be acceptable and is attractive where standard LA 3.0 recordings are routine. Recent comparative work suggests both RB and WW can achieve useful diagnostic accuracy when implemented thoughtfully [3].

### 4.2. Electrodes and Instrumentation

DTL (or gold foil) corneal electrodes maximize signal-to-noise, but modern skin (sensor-strip) electrodes used by handheld systems (e.g., RETeval) have improved feasibility without mydriasis and support clinic-ready PhNR protocols. Skin electrodes produce lower amplitudes and SNR than DTL and may slightly alter implicit times; laboratories should maintain separate normative ranges and be aware of the trade-offs [6,7].

Recent developments in recording methodology have introduced novel electrode configurations for PhNR acquisition, with studies demonstrating that alternative electrode systems can provide reliable and clinically feasible PhNR recordings, potentially improving patient comfort and signal accessibility [8].

### 4.3. Pupil Size, Retinal Illuminance, and Mydriasis

Handheld systems compensate stimulus strength by pupil-tracked Trolands, making many protocols reliable without dilation. That said, very large pupils, the Stiles-Crawford effect, or atypical optics can still introduce variability, and some studies show modest PhNR amplitude dependencies on pupil size, particularly at weaker flashes, underscoring the value of stable test conditions [5].

### 4.4. PhNR Measurement and Selection of PhNR1 Versus PhNR2

PhNR amplitude is typically quantified using trough-based measurements relative to the baseline or b-wave peak, as specified above. When both PhNR1 and PhNR2 components were identifiable, PhNR2 was selected as the primary outcome measure, defined as the later negative trough following the b-wave, provided that it demonstrated consistent latency and reproducible amplitude across repeated recordings. In cases where PhNR2 was not clearly defined, including shallow or absent second troughs or poor inter-trial reproducibility, PhNR1 was used as an alternative metric. To ensure consistency, the selected trough (PhNR1 or PhNR2) was applied uniformly within each subject, and waveform quality was confirmed through averaging of repeated sweeps where feasible. The choice of metric was documented for each recording to support reproducibility and facilitate comparison across datasets.

### 4.5. Multifocal PhNR (mfPhNR)

mfPhNR extends the concept to topographically map RGC function across the posterior pole. Optimized “fast” protocols can enhance glaucoma detection, particularly for early/suspect damage, and provide spatial complementarity to OCT and visual fields. Electrode choice and dilation state influence mfPhNR SNR and implicit times; DTL is preferred when feasible [9].

## 5. Applications of Photopic Negative Response

### 5.1. Glaucoma: The Flagship Indication

#### 5.1.1. Diagnostic Utility

**Figure 2:**
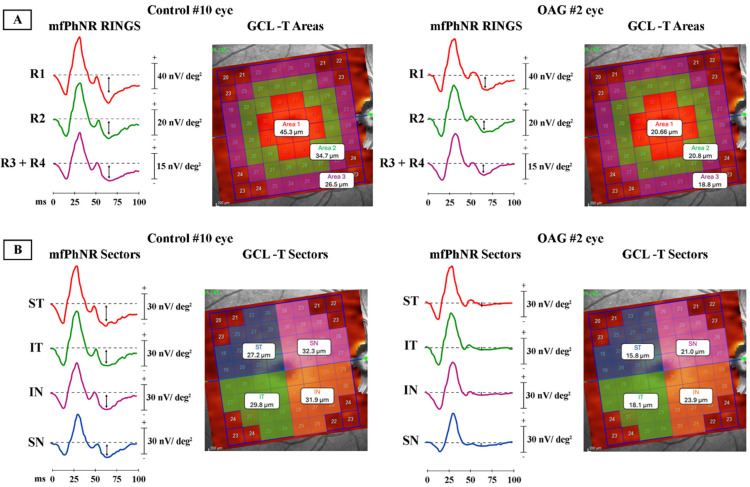
Functional and structural assessment of retinal ganglion cells (RGCs) using multifocal photopic negative response (mfPhNR) and ganglion cell layer thickness (GCL−T) in a control eye (#10) and an open−angle glaucoma eye (OAG#2). (**A**) mfPhNR ring analysis and corresponding optical coherence tomography (OCT) regions. (**B**) mfPhNR sector analysis and corresponding OCT sectors. Across both analyses, the OAG eye shows reduced mfPhNR responses and decreased GCL−T compared with the control eye [44].

Recent studies from broader clinical journals further support the role of ERG biomarkers in glaucoma, demonstrating that PhNR and related ERG features provide clinically meaningful functional information that complements structural imaging and perimetry, particularly in early or preperimetric glaucoma. As the most extensively studied and clinically mature application of PhNR, glaucoma serves as a representative model of retinal ganglion cell dysfunction and is therefore discussed in greater depth below. PhNR reduction is a hallmark in POAG and ocular hypertension with early neuroretinal compromise. Correlations between PhNR and OCT measures, including retinal nerve fiber layer (RNFL), ganglion cell-inner plexiform layer (GCIPL), and RGC, as well as perimetry indices are robust across devices and cohorts (Figure 2). Inter-eye analyses show that the eye with thinner RNFL typically has a lower PhNR amplitude, even at early stages, suggesting utility in asymmetric disease [10,11]. Comparative work indicates that both RB and WW PhNR protocols can identify RGC dysfunction with diagnostic accuracy overlapping other established tests; the choice of protocol should reflect local workflows and the ability to ensure stable, repeatable signals. Systematic reviews emphasize the synergy of combining ERG markers (PhNR + photopic markers beyond PhNR) with structural features for optimal staging [12,13]. Clinical studies using handheld and conventional ERG systems have demonstrated that PhNR provides reproducible diagnostic performance in detecting glaucomatous RGC dysfunction [14].

#### 5.1.2. Monitoring Disease Progression

PhNR reflects glaucomatous dysfunction and correlates with disease severity, but its high test–retest variability limits its value for tracking progression in clinical practice. Longitudinal studies indicate that OCT parameters, particularly RGC and circumpapillary retinal nerve fiber (cpRNFL), provide more reliable measures of progression, while PERG may add complementary functional information. Thus, PhNR is more suited for early detection or cross-sectional assessment than for routine monitoring of longitudinal change [15]. Recent evidence further supports the relationship between PhNR and structural measures of retinal integrity, demonstrating significant associations between PhNR parameters and OCT-derived metrics, reinforcing its role as a functional correlate of RGC damage [16,17].

#### 5.1.3. Treatment Response and Reversibility

One of the most clinically meaningful attributes of the PhNR is its potential sensitivity to short-term functional improvement after intraocular pressure (IOP) reduction. In surgical contexts, such as trabeculectomy or filtration procedures, PhNR amplitudes have been shown to increase within days, presumably reflecting early recovery of RGC function before structural restitution occurs. Similar trends have been reported following medical IOP lowering in ocular hypertension and early glaucoma. However, recent prospective data with preservative-free latanoprost demonstrated significant IOP lowering without consistent group-level changes in PhNR over three months, highlighting variability and the need for longer follow-up [18,19]. Importantly, observed short-term improvements in PhNR following IOP decrease should be understood as evidence of potentially reversible dysfunction at the group level, rather than as a precise biomarker for individual longitudinal monitoring, given known test–retest variability. This responsiveness nonetheless underscores PhNR’s value as an objective indicator of early therapeutic effect and as a supportive tool for patient counseling.

Although several studies suggest that PhNR may reflect reversible RGC dysfunction following intraocular pressure (IOP) reduction, more recent prospective data have reported inconsistent findings. These discrepancies may be attributed to differences in sample size, disease stage, duration of follow-up, and type of therapeutic intervention. In particular, smaller cohorts and short follow-up periods may limit the ability to detect subtle functional recovery. Additionally, variability in recording protocols and outcome metrics may further contribute to inconsistent results. Therefore, while PhNR shows potential as a marker of treatment-related functional change, its clinical utility in this context remains to be fully established.

These findings are supported by recent interventional data demonstrating that nicotinamide supplementation can enhance PhNR amplitudes in normal-tension glaucoma, suggesting that PhNR reflects, at least in part, reversible RGC dysfunction rather than solely irreversible structural loss [20]. Recent longitudinal evidence in patients with pituitary macroadenoma further indicates that PhNR alterations reflect dynamic changes in RGC function, correlating with structural OCT measures during disease course, thereby supporting its role in monitoring functional recovery following treatment [21].

#### 5.1.4. Clinical Implementation Considerations

For glaucoma clinics, a pragmatic pathway is: (1) baseline PhNR at diagnosis/suspicion alongside OCT and perimetry; (2) post-intervention PhNR to document functional response; (3) periodic follow-up every 6–12 months (or sooner in high-risk eyes) using the same device, electrode type, and stimulus protocol; (4) combine trend-based PhNR metrics with OCT thinning and SAP event/progression analyses to decide on therapy escalation.

### 5.2. Neuro-Ophthalmology: Optic Nerve Disorders Beyond Glaucoma

Abnormal PhNR has been consistently observed across a spectrum of optic nerve disorders, supporting its role as a global marker of RGC dysfunction [14].

#### 5.2.1. Optic Neuritis and Demyelinating Disease

In both acute and previous optic neuritis, PhNR amplitudes are diminished, indicating axonal dysfunction or loss [22,23]. Comparative studies with PERG suggest that the two techniques provide complementary insights, while the ability to record PhNR using modern handheld systems without pharmacological dilation offers a practical advantage for acutely ill patients or those with photophobia [24].

#### 5.2.2. Ischemic Optic Neuropathy (NAION) and Traumatic Optic Neuropathy (TON)

PhNR is markedly attenuated in ischemic and traumatic optic neuropathies, consistent with axonal injury, and helps distinguish RGC-specific dysfunction from outer retinal or macular disease. Using the handheld RETeval system, Yamashita et al. [14] demonstrated that PhNR amplitudes were significantly reduced across a spectrum of optic nerve disorders, and that their diagnostic performance (AUC 0.78–0.86) was comparable to OCT-derived circumpapillary RNFL thickness (AUC 0.76–0.99). Importantly, correlations between PhNR and cpRNFLT (Circumpapillary Retinal Nerve Fiber Layer Thickness) were most evident in acute ON/ION and TON/DOA, while weaker in chronic disease. These findings highlight PhNR’s value as an accessible functional biomarker, particularly when OCT interpretation is limited by media opacity or disc swelling.

#### 5.2.3. Compressive Optic Neuropathy (Chiasmal/Pituitary)

Preoperative PhNR reduction is associated with visual field loss, and early postoperative improvements may parallel successful decompression. Evidence from optic chiasmal decompression shows that visual field recovery occurs rapidly within the first few months, accompanied by progressive thinning of the RGC and RNFL despite functional gains, underscoring the dissociation between structure and function. Although the evidence base is smaller than in glaucoma, both mfPhNR and full-field PhNR (ffPhNR) provide objective indices of RGC function when SAP or OCT results are equivocal or affected by artifacts, and representative studies indicate their potential prognostic utility after decompression [25].

#### 5.2.4. Hereditary Optic Neuropathies

In Leber hereditary optic neuropathy (LHON), PhNR amplitudes are markedly reduced in affected eyes and show mild reductions in asymptomatic carriers, reflecting subclinical RGC dysfunction and highlighting PhNR’s potential for monitoring at-risk individuals or evaluating treatment effects in clinical trials [14,26].

#### 5.2.5. Idiopathic Intracranial Hypertension (IIH)

Several key studies have evaluated PhNR in IIH. Moss, Park, and McAnany provided the earliest and most influential evidence, demonstrating that IIH patients exhibited significantly reduced PhNR amplitudes compared with age-matched controls [27]. Importantly, PhNR amplitude correlated with visual field mean deviation, OCT retinal nerve fiber layer thickness, and ganglion cell complex metrics. This study established that PhNR carries clear structure-function relevance and may detect RGC injury that is otherwise difficult to identify when optic disc edema is present. Subsequent work by Park and colleagues compared the PhNR with the pattern electroretinogram (pERG) in IIH and showed that the two tests provide complementary information. Whereas the pERG, which reflects macular RGC function, demonstrated strong correlations with HVF MD and ganglion cell complex volume (GCCV), the ffPhNR identified abnormal global RGC activity in a substantial subset of patients, despite the overall group mean reduction approaching but not always reaching statistical significance in all measures. In contrast, the focal PhNR (fPhNR) was largely within normal limits and showed no significant structure-function associations. Together, these findings indicate that pERG and ffPhNR capture different aspects of RGC dysfunction in IIH, and their combined use may enhance electrophysiologic evaluation in this condition [28].

More recent research has explored the feasibility of using portable, handheld devices to record PhNR in clinical settings beyond the electrophysiology laboratory. Raharja et al. [29] demonstrated that PhNR measurement with handheld ERG device is feasible in IIH patients, correlates moderately with OCT parameters, and could allow rapid monitoring of RGC function even in emergency departments, weight-loss clinics, or inpatient settings. While variability remains higher than in standard ERG systems, this line of research represents an important step toward broader accessibility. Ongoing prospective studies, particularly those based in the United Kingdom, are now evaluating PhNR in pediatric IIH and other causes of raised intracranial pressure. These protocols emphasize shorter testing durations and child-friendly stimulus paradigms, with the goal of creating a reliable, objective biomarker for early detection and longitudinal monitoring of RGC dysfunction in pediatric populations.

Clinically, PhNR offers several advantages in managing IIH. It provides an objective, reproducible measure of RGC integrity that is independent of patient performance, in contrast to visual fields. It may detect functional deficits earlier than standard tests and can help distinguish reversible dysfunction associated with acute swelling from permanent axonal loss. PhNR can also help clarify discordances between visual fields and OCT, particularly in patients whose swollen optic discs obscure underlying ganglion cell loss. Because it directly assesses function, PhNR can also be used to monitor treatment response, whether to weight loss, acetazolamide therapy, optic nerve sheath fenestration, or cerebrospinal fluid diversion procedures [30]. PhNR abnormalities reflect dysfunction of RGCs secondary to systemic neurological or metabolic stress, reinforcing the concept of the retina as a functional biomarker of extra-ocular disease. However, current evidence remains limited by relatively small cohort sizes and heterogeneity in study design, and further longitudinal studies are needed to define its role in routine clinical monitoring.

### 5.3. Inner Retinal Ischemia and Vascular Disease

#### 5.3.1. Central Retinal Artery Occlusion (CRAO)

Central retinal artery occlusion acutely damages the inner retina, producing pronounced ERG abnormalities. PhNR amplitudes are often markedly reduced or extinguished and correlate closely with the severity of ischemia [31]. When combined with other ERG parameters such as the B wave amplitude or B/A ratio, PhNR contributes to a multiparameter profile that reflects baseline retinal dysfunction and predicts subsequent improvements in visual acuity and visual fields. These findings underscore its value for prognostication and patient counseling in CRAO [32].

#### 5.3.2. Diabetic Retinopathy (DR) and Diabetes Without Retinopathy

Diabetes can affect the inner retina early, often preceding ophthalmoscopically detectable retinopathy. PhNR amplitudes are reduced in patients with diabetes both with and without clinically evident diabetic retinopathy, supporting the concept of diabetic retinal neuropathy [33]. Incorporating PhNR into risk stratification may help guide referral and follow-up intervals, particularly for patients with subtle imaging findings or symptoms disproportionate to fundus appearance. Representative cohort studies and reviews summarize these trends [24].

#### 5.3.3. Full-Thickness Macular Hole and Surgical Monitoring

ffERG, including PhNR, has also been evaluated in localized macular pathology. In patients with full-thickness macular hole (FTMH) undergoing surgical repair, no significant preoperative differences in ERG parameters, including PhNR, were observed between affected and fellow eyes. Nevertheless, PhNR-based assessment may still provide a non-invasive tool for longitudinal monitoring of retinal function following surgical intervention, supporting its potential utility in tracking postoperative retinal recovery [34].

#### 5.3.4. Rare Genetic Disorders and Syndromic Associations

The applicability of the PhNR extends beyond common retinal and optic nerve diseases to rare genetic and systemic disorders. A recent case of geleophysic dysplasia associated with an *FBN1* mutation demonstrated markedly reduced PhNR amplitudes, consistent with RGC dysfunction. Notably, these electrophysiological findings were observed in the presence of optic disc abnormalities, highlighting the ability of PhNR to detect inner retinal dysfunction even in uncommon multisystem conditions. In the largest ERG study conducted in congenital PAX6-related aniridia, reduced W-ratios indicated impaired postreceptoral signal transmission, with further reductions observed in patients with comorbid glaucoma, and recordings using the handheld RETeval system were well tolerated across a wide age range [35]. Although evidence remains limited, such observations support the broader utility of PhNR as a functional biomarker across a wide spectrum of RGC pathologies [36].

### 5.4. Pediatric and Special Populations

Children and patients who cannot tolerate corneal electrodes can still undergo PhNR recording using skin electrodes with handheld devices. Although skin electrodes yield smaller amplitudes and lower signal-to-noise ratios, this compromise is often acceptable when age-appropriate normative data are applied and technicians emphasize patient comfort and fixation. In neuro-pediatric settings, PhNR recordings are particularly valuable when visual field testing is unfeasible and OCT acquisition is limited [7,37].

## 6. PhNR Complements Other Tests

### 6.1. Optical Coherence Tomography and Optical Coherence Tomography Angiography

PhNR amplitudes correlate closely with RNFL and macular GCIPL/RGC thickness in glaucoma and other optic neuropathies [38]. Nevertheless, structure-function discordance is common in neuro-ophthalmology, and PhNR provides an objective functional measure that can clarify cases with ambiguous OCT findings, such as those complicated by edema, disc tilt, or high myopia. Intereye comparisons are particularly valuable in asymmetric disease, reflecting differential structural and functional involvement [11,39].

According to the study of Tabl et al. [40], there is a clear and clinically meaningful correlation between PhNR and optical coherence tomography angiography (OCTA) parameters in glaucoma. Specifically, OCTA-derived vascular measures such as peripapillary and macular vessel density show a significant positive correlation with PhNR amplitude and a negative correlation with PhNR implicit time, meaning that reduced retinal microvascular perfusion is associated with decreased RGC function and delayed electrophysiological response. As glaucoma severity increases, both OCTA vessel density and PhNR amplitude decline in parallel, indicating that microvascular compromise and functional loss of ganglion cells are closely linked processes.

### 6.2. Visual Field Testing

While perimetry remains the gold standard for assessing functional impairment in glaucoma, it is inherently subjective and prone to variability [41]. PhNR provides an objective measure of RGC function, making it particularly useful in patients with unreliable visual fields, cognitive impairment, or suspected malingering. When used alongside standard automated perimetry, PhNR can enhance confidence in detecting disease progression, particularly when both modalities show concordant changes [15].

### 6.3. PERG and Other ERGs

PERG, driven by patterned stimuli, and PhNR, elicited by flashes, both assess overlapping RGC function through distinct input pathways and circuits. In clinical practice, PERG may be slightly more sensitive to macular RGC dysfunction, whereas PhNR is easier to administer and more resilient to optical blur or media opacity [42]. When feasible, combining PERG and PhNR can enhance detection of inner-retinal dysfunction across conditions such as idiopathic intracranial hypertension and early glaucoma [24]. A novel complementary measure, the mesopic negative response (MeNR), has recently been described as a marker of RGC function within the rod pathway, recordable without dark adaptation using silent substitution. In severe glaucoma, MeNR amplitude was significantly attenuated and strongly correlated with PhNR amplitude (r = 0.86), suggesting it may provide additional functional information about RGC integrity through a distinct pathway [43].

## 7. mfPhNR and Topography

mfPhNR (Figure 2) bridges global ERG measures and localized retinal dysfunction, with fast stimulation protocols improving sensitivity for detecting early or suspect glaucoma [44]. Its topographic patterns often correspond to arcuate or hemifield defects observed on perimetry and macular OCT, supporting its role in pre-perimetric assessment and evaluation of treatment-related functional changes [9].

mfPhNR response amplitude density (nV/deg^2^) was quantified as the baseline-to-trough amplitude within a defined post-stimulus time window. Spatial analysis included both concentric ring-based averaging across 0–20° of eccentricity (central 0–5°, parafoveal 5–10°, and peripheral 10–20° regions) and sector-based analysis of the 5–20° annulus, divided into superior temporal, inferior temporal, inferior nasal, and superior nasal quadrants.

Corresponding structural measurements were obtained using optical coherence tomography (OCT), with ganglion cell layer thickness (GCL-T) quantified in matched macular regions for both ring-based and sector-based analyses, enabling structure-function comparison.

## 8. Implementation Guide: From Norms to Reporting

### 8.1. Building Reliable Norms

Establish age-matched, device- and electrode-specific reference ranges; account for pupil management (dilated vs. nondilated) and stimulus family (RB vs. WW). Report which metric (BT/PT/ratio) you use and stick with it across visits [3].

### 8.2. Standardization of Acquisition

Use the same electrode type each visit (ideally DTL for maximal SNR, skin for tolerance/feasibility) and the same adaptation, flash strength, and background. Note media status and fixation quality; repeat noisy traces [7].

### 8.3. Select Clinically Meaningful PhNR Metrics

In standard glaucoma or optic neuropathy practice, baseline-to-trough (BT) or peak-to-trough (PT) amplitudes are straightforward and interpretable. Normalized ratios may be useful when absolute amplitudes vary, but they do not necessarily outperform raw amplitudes—especially in myopic patients. Local validation is recommended before routine use [45].

### 8.4. Integration of PhNR with Structural Imaging

Generate a structured report including PhNR amplitude (with z-scores relative to normative data) and OCT RNFL and GCIPL measurements. Highlight any discordance between modalities and recommend repeat or alternative assessments when indicated [46].

### 8.5. Monitoring of Longitudinal Changes Using Consistent Recording Setups

Early post-treatment improvements in PhNR amplitude can help guide patient counseling and adherence discussions. For disease progression, evaluate trends across serial PhNR recordings and, when available, complementary structural and functional measures, rather than relying on single visit thresholds [18].

## 9. Special Topics and Emerging Directions

### 9.1. Protocol Harmonization and RB vs. WW

Red-on-blue (RB) stimuli generally demonstrate higher sensitivity for detecting RGC dysfunction, whereas white-on-white (WW) PhNR recorded using the standard LA 3 photopic ERG offers comparable specificity and can be implemented with minimal protocol modification. At present, both approaches are used in research and selected clinical settings, but no consensus standard has been established. Ongoing head-to-head comparisons and multicenter harmonization studies are expected to clarify optimal protocols for different clinical applications [12].

### 9.2. Handheld, Mydriasis-Free ERG in Triage

Pupil-tracked Troland stimuli and skin electrodes enable rapid PhNR assessment in settings where OCT or perimetry may be unavailable (e.g., suspected optic neuritis or CRAO/CRVO triage). These systems are clinically available and feasible for point-of-care use [47]. However, their diagnostic performance remains less well validated than conventional ERG. Awareness of limitations related to pupil-size compensation and signal-to-noise ratio is essential.

### 9.3. Beyond the Trough: Richer Photopic Features

Machine learning studies suggest that additional photopic ERG parameters, including a-wave, b-wave, and i-wave amplitudes, provide complementary information about RGC function and glaucoma severity. At present, these approaches remain investigational and are not part of routine clinical workflows. Multifeature models may ultimately outperform single-metric PhNR, but require external validation, standardization, and prospective evaluation before clinical adoption [13].

### 9.4. Multifocal PhNR Analytics

Multifocal PhNR enables spatially resolved assessment of RGC function and is primarily used in research settings. Advanced analytical approaches, such as time–frequency decomposition, may improve sensitivity in ischemic conditions such as central retinal vein occlusion. However, these techniques are not yet standardized and have limited clinical availability. Integration with OCT angiography represents a promising direction for future multimodal assessment of neurovascular compromise [9,48].

### 9.5. PhNR for Early Functional Recovery Assessment

The PhNR can improve within days following intraocular pressure reduction, supporting its role as a potential early functional biomarker of neuroretinal recovery. Currently, this application is best considered a research endpoint rather than an established clinical tool. Incorporation into clinical trials may enable shorter study durations or smaller sample sizes, but further validation is required before routine clinical use [18].

### 9.6. Clinical Role of PhNR

Although numerous studies report associations between PhNR amplitude, structural loss, and visual field indices, these relationships should be interpreted as contextual rather than deterministic. In clinical practice, PhNR does not replace OCT or perimetry, nor does it reliably track long-term progression. Instead, its current clinical value lies in providing an objective snapshot of RGC function, particularly in early disease, asymmetric cases, or when psychophysical testing is unreliable.

## 10. Limitations and Caveats

Despite its promise, several barriers limit the widespread clinical adoption of PhNR, including lack of normative harmonization across platforms, electrode-dependent variability, modest test–retest repeatability, and limited availability in routine clinical settings.

### 10.1. Signal Dependence and Variability

PhNR is sensitive to electrode type, stimulus details, and analysis choices. Cross-platform amplitudes are not interchangeable; laboratories must avoid mixing norms [7].

### 10.2. Media, Refractive Status, and Myopia

Although less sensitive to blur than PERG, cataract and severe dry eye can still degrade signal-to-noise ratio. Myopia may alter diagnostic cut-offs and reduce the utility of certain normalization ratios versus raw amplitude [45].

### 10.3. Physiological Specificity

PhNR is predominantly reflective of RGC function; however, it is not entirely specific. Conditions such as severe inner retinal ischemia or widespread retinal diseases can influence PhNR measurements, potentially confounding interpretations. Therefore, integrating multimodal assessments including OCT, standard automated perimetry, and mfPhNR is recommended to enhance diagnostic accuracy and clinical decision making [46].

### 10.4. Norms and Age Effects

Age effects are modest but real; use age-matched, device-specific norms [3].

## 11. Future Directions

### 11.1. Stimulus Selection and Standardization in PhNR Recordings

The study found that red-on-blue (RB) stimuli for PhNR provided slightly higher sensitivity than white-on-white (WW) stimuli, while specificities were comparable. The authors note that WW PhNR can be integrated with minimal protocol changes, supporting flexibility across laboratories. These findings highlight the potential value of harmonizing stimulus protocols and metrics in multi-center studies to improve comparability of results [12].

### 11.2. Deeper Feature Extraction

Incorporating additional parameters from the electroretinogram (ERG), such as amplitudes of the a-, b-, and i-waves, alongside the trough between the b- and i-waves, provides a more comprehensive assessment of RGC function. Utilizing machine learning models, specifically multivariate adaptive regression splines (MARS), with these composite photopic markers enhances the prediction of glaucoma severity compared to relying solely on the PhNR amplitude. This approach offers improved sensitivity for early detection and more nuanced staging of the disease [13].

### 11.3. Combining mfPhNR with OCT/OCTA and Perimetry

Combining mfPhNR with OCT, optical coherence tomography angiography, and standard automated perimetry can provide a comprehensive assessment of RGC function and vascular health. This multimodal approach enhances the detection of localized deficits and may offer individualized neurovascular risk profiles in glaucoma and ischemic retinal diseases [9,48].

### 11.4. Portable Electrophysiology for Rapid PhNR Assessment

Handheld electroretinography (ERG) devices that utilize pupil-tracking technology and do not require mydriasis offer the potential to bring PhNR testing to settings such as emergency departments, community screening programs, and longitudinal tele-ophthalmology consultations. However, it is essential to implement robust quality control measures and establish normative data specific to these portable devices to ensure accurate and reliable results [5,14].

For practical clinical reference, a comparative summary of PhNR alongside optical coherence tomography (OCT), visual field testing, and pattern electroretinography (PERG), including their respective strengths, limitations, and primary Clinical Role, is provided in Table 2.

To clarify the available evidence, the clinical utility of the PhNR can be framed across three domains: (1) cross-sectional functional assessment, where evidence is strongest; (2) short-term responsiveness to therapeutic interventions, where PhNR may detect reversible dysfunction; and (3) longitudinal progression monitoring, where its role is limited by variability and methodological heterogeneity.

## 12. Conclusions

The PhNR has demonstrated promising and context-dependent value as a complementary functional biomarker with strongest evidence in selected clinical contexts rather than universal clinical adoption. It offers an objective measure of RGC function that correlates with structural and functional indices, with particular value when visual fields are unreliable, OCT findings are ambiguous, or rapid functional changes require documentation. When acquired using standardized ISCEV extended protocols, paired with careful metric selection, and thoughtfully integrated with OCT and perimetry, PhNR enhances diagnostic confidence, guides therapeutic decisions, and informs patient counseling across a broad spectrum of optic nerve and inner retinal disorders. Looking forward, harmonized protocols, topographic and multimodal integration, and advanced analytical approaches promise to further expand its clinical utility and impact. At present, the most evidence-supported clinical utility of PhNR lies in complementary functional assessment, early detection of RGC dysfunction, and short-term evaluation of treatment response, rather than routine longitudinal monitoring. Notably, findings across studies are not entirely consistent. Variability in study design, patient populations, recording protocols, and analytical methods contributes to heterogeneity in reported PhNR outcomes. In particular, differences in stimulus parameters, electrode types, and amplitude metrics can affect both sensitivity and reproducibility, leading to discrepancies in diagnostic performance and longitudinal assessment. While many studies support the utility of PhNR in detecting RGC dysfunction, others report more modest or variable associations, especially in longitudinal monitoring. These inconsistencies highlight the need for further standardization and larger, well-controlled studies to better define its clinical role.

## Figures and Tables

**Table 1:** Key physiological and methodological features of the photopic negative response (PhNR).

Domain	Key Points	Clinical Implications
Primary Generators	Originates predominantly from retinal ganglion cells (RGCs) and their axons; selectively reduced with RGC injury.	Reflects RGC function; supports early detection of ganglion cell damage.
Waveform Components	Negative trough following the cone-driven b-wave; measured as BT (baseline-to-trough) or PT (peak-to-trough).	Helps distinguish inner retinal vs. outer retinal dysfunction.
Amplitude Metrics	BT yields higher variability; PT correlates more strongly with other ERG components; ratios to b-wave reduce variability but may obscure disease changes.	Influences sensitivity, reproducibility, and inter-session comparability.
PhNR1 vs. PhNR2	Trough may occur before or after the i-wave; both are strongly correlated; PhNR1 can be used when the i-wave obscures PhNR2.	Choice affects stability vs. sensitivity of measurements.
Age Effects	Modest age-related decline, and age-matched norms required.	Requires age-adjusted interpretation and normative data.
Influence of Stimulus Design	Red-on-blue (RB) generally yields higher sensitivity; white-on-white (WW) is clinically practical and harmonizable across centers.	Affects signal quality, diagnostic performance, and cross-study consistency.

**Table 2:** Comparison of PhNR with other clinical modalities.

Modality	Strengths	Limitations	Most Likely Clinical Role
OCT	High-resolution structural assessment	Limited functional information	Diagnosis and structural monitoring
Visual Fields	Direct functional relevance	Subjective, variable	Functional assessment and progression
PERG	Sensitive to macular RGC function	Limited availability, technically demanding	Early dysfunction detection
PhNR	Objective global RGC function	Variability, limited longitudinal reliability	Complementary biomarker, early detection, treatment response

## Data Availability

All data supporting this study are publicly available from the original publications included in the review. References to these studies are provided in the manuscript.
